# Estimating rates of de novo infection and mitotic replication in HTLV-1 persistence: de novo infection continues after early infection

**DOI:** 10.1186/1742-4690-12-S1-P46

**Published:** 2015-08-28

**Authors:** Daniel J Laydon, Vikram Sunkara, Charles RM Bangham, Becca Asquith

**Affiliations:** 1 Department of Medicine, Imperial College London, UK

## 

Human T-lymphotropic virus type-1 (HTLV-1) persists within hosts via de novo infection (infectious spread) and infected cell proliferation (mitotic spread), creating a population structure of multiple clones (infected cell populations with identical genomic proviral integration sites). However, the relative contributions of these mechanisms are unknown, and will determine the efficacy of antiretroviral therapy. The prevailing view is that infectious spread does not contribute to HTLV-1 proviral load after early infection. However, this notion is based on low-throughput and imprecise data on clonal integration site abundance and diversity. We recently used our high-throughput protocol that maps and quantifies the abundance of HTLV-1 integration sites to estimate a diversity of between 10^4 and 10^5 HTLV-1 T cell clones in the body of an asymptomatic carrier or patient with HAM/TSP. Since infectious spread establishes new clones, high clonal diversity implies substantial infectious spread. HTLV-1 clonal abundance varies within individual hosts by several orders of magnitude. Therefore quantifying within-host HTLV-1 dynamics requires mathematical modelling at multiple scales, and stochastic processes are necessary to describe the behaviour of small clones. Here we apply a hybrid model of deterministic and stochastic processes to time-series patient data, to estimate the relative contributions of infectious spread and mitotic spread. We find, contrary to previous belief that infectious spread persists during chronic infection, even after the proviral load has reached its set point. The risk of HTLV-1-associated malignant and inflammatory disease is strongly correlated with the proviral load: the load in turn is correlated with the total number of HTLV-1-infected clones, but not with the degree of oligoclonal proliferation. Our results therefore suggest that attempts to suppress de novo infection may reduce the risk of malignant transformation.

